# Erectogenic and Aphrodisiac Effects of *Butea frondosa* Koenig ex Roxb. in Rats: Involvement of Enzyme Inhibition

**DOI:** 10.1155/2013/874894

**Published:** 2013-08-24

**Authors:** Sumanta Kumar Goswami, Mohammed Naseeruddin Inamdar, Manoj Kumar Pandre, Rohitash Jamwal, Shekhar Dethe

**Affiliations:** ^1^Department of Pharmacology, Al-Ameen College of Pharmacy, Near Lalbagh Main Gate, Hosur Road, Bangalore 560027, India; ^2^Bioassay Lab, Research and Development Centre, Natural Remedies Pvt. Ltd., Veerasandra Industrial Area, 19th KM Stone, Hosur Road, Electronic City, Bangalore 560100, India

## Abstract

*Butea frondosa* Koenig ex Roxb. (BF) is traditionally used to manage male sexual disorders including erectile dysfunction (ED). Methanol extract of BF (bark) inhibited Rho-kinase 2 (ROCK-II) enzyme activity *in vitro* with an IC_50_ of 20.29 ± 1.83 **μ**g/mL. The relaxant effect of methanol extract of BF (MEBF) was studied on phenylephrine precontracted corpus cavernosum smooth muscle (CCSM) isolated from young rats. The effect of MEBF treatment on sexual behaviour of both young (5 month) and aged (24 month) rats was also studied in addition to the influence on smooth muscle, collagen (collagen-I and -III) level in penis, and sperm characteristics of young and aged rats. MEBF relaxed CCSM up to 21.77 ± 2.57% and increased sexual behavior of young and aged rats. This increase in sexual function could be attributed to ROCK-II inhibition and increase in ratio of smooth muscle to collagen level in rat penile tissue. Increased sperm production and decreased defective sperms in young and aged rats corroborate the usefulness of *Butea frondosa* in male infertility in addition to ED.

## 1. Introduction

Male sexual dysfunction (MSD) is defined as difficulty of male partner to have sexual activity with female partner, and it affects male population of different ages, ethnicities, and cultural backgrounds. The MSD includes lack of sexual desire, disorder of orgasm, erectile dysfunction, disorder of ejaculation, and long lasting priapism [1]. Erectile dysfunction (ED), the most prevalent MSD, is the inability of male partner to achieve and maintain an erect penis of adequate rigidity for sexual intercourse [2]. Penile erection is centrally regulated by mediators, namely, dopamine, noradrenaline, acetylcholine, nitric oxide (NO), and so forth, as well as peripherally by neurotransmitters (noradrenaline, acetylcholine, etc.), second messengers (cGMP, cAMP, etc.), enzymes (Nitric oxide synthases/NOSs, soluble guanyl cyclase/sGC, phosphodiesterases/PDE, etc.), and ion channel [3, 4]. 

Some major factors reported to cause ED include ageing, atherosclerosis, depression, and renal failure. A degree of ED also develops due to use of thiazide diuretics and *β*-blockers. Phosphodiesterases type 5 (PDE-5) inhibitors such as sildenafil, tadalafil, and vardenafil are generally used for the management of ED [5–7]. 

In addition to synthetic drugs, herbal extracts have been reported for their erectogenic potential [1, 8, 9], and Indian medicinal plants have been used to manage ED since times immemorial. “Vajikarana chikitsa” is a branch of Ayurveda, Indian system of alternate medicine, that describes different herbal formulations for management of male sexual disorders including ED. “Vajikarana” herbs/aphrodisiacs are the herbs that have been used in the Ayurvedic system of medicine to treat ED. *Butea frondosa* has been enlisted in Ayurveda as “Vajikarana” herb for the management of ED [10]. 

*Butea frondosa* Koenig ex Roxb./*Butea monosperma* (Lamk.) Taub. (family: Fabaceae) is a long and deciduous tree, commonly distributed throughout India. Classical names of the plant are Raktapushpaka, Palasha, Kinshuka, Parna, and so forth, and it is identified by leaves that are long petiolated, 3-foliate, flowers that are bright orange red coloured (hence the name raktapushpaka, meaning the plant bearing blood-red coloured flower), and large and in rigid recemes. Many parts of the plant like bark, leaf, flower, and gum are useful as per Ayurvedic literature and have been reported to have aphrodisiac properties [11]. Even though a study on the aphrodisiac activity of *Butea frondosa* (BF) bark extract is available, there is no existing evidence to elucidate the mechanism of action [9].

Decrease in mount latency (ML) implies increase in sexual urge that is controlled by central nervous system (CNS) mediated action. Increased erectile function is implicated by an increase in intromission that is controlled peripherally by NO, second messengers, and enzymes [3, 12] such as ROCK-II, as ROCK-II inhibitors are reported to increase erectile function [13, 14]. In our earlier study, methanol and successive aqueous extracts of BF were reported to inhibit ROCK-II [15]. In the present study, ROCK-II inhibition study was performed to determine IC_50_ of methanol extract of *Butea frondosa *(MEBF), while relaxant effect of extract was studied on phenylephrine precontracted isolated rat corpus cavernosum smooth muscle/CCSM [15, 16]. The present study is an attempt to identify a possible mechanism of action for the reported efficacy of the plant. In addition, effect of herbal extract was also studied on sexual activity [1, 8, 9], sperm count/characteristics [17–19], and ratio of smooth muscle:collagen level in rat penile tissue [20–23] of young (5 months) and aged rats (24 months). 

## 2. Materials and Methods

### 2.1. Plant Materials and Extraction

The stem bark of *Butea frondosa* was collected by Natural Remedies Private Limited (NRPL) and authenticated by Dr. P. Santhan (Botanist), and a specimen sample was stored in NRPL repository with specimen number NPL/CD/95. Methanol extract of *Butea frondosa* bark was prepared as mentioned elsewhere. Briefly, freshly collected *Butea frondosa* bark was dried under shade, and 500 g of this bark was powdered and extracted with 1.5 L of methanol under reflux on a water bath maintained at 60 ± 1°C for 90 minutes and then filtered. Marc was extracted twice, with fresh solvent every time, and the combined filtrate was concentrated under 400 mm Hg vacuum at 60 ± 1°C to obtain the methanol extract of *Butea frondosa* (MEBF) [15].

### 2.2. Animals

Wistar rats of either sex maintained at 25 ± 2°C were used for the study and were provided with rat chow and drinking water *ad libitum*. Young rats (5 months) weighed 200–230 g, while aged rats (24 months) weighed 290–320 g. The animal experimentation was performed as per *Guide for the Care and Use of Laboratory Animals *of the National Institutes of Health and guidelines set by CPCSEA (India). Experiment protocol was reviewed and approved by Animal Ethics Committee of Al-Ameen College of Pharmacy, Bangalore, India.

### 2.3. Chemicals and Materials

Homogeneous time resolved fluorescence (HTRF) KinEASE STK S2 Kit (Cisbio Bioassays, France), Rho-kinase 2 active (Upstate, USA), ROCK-II inhibitor Y-27632 (CALBIOCHEM, USA), 384-well low volume black plate (Greiner, USA), Ketamine hydrochloride (Neon Laboratories Ltd, India), Xylazine (Indian Immunologicals Ltd, India), diethylstilbestrol (Penta Pharmaceuticals, India), and progesterone (Sun Pharmaceutical Ind. Ltd., India) were procured. Adenosine triphosphate/ATP, triton X-100/4-(1,1,3,3-tetramethylbutyl)phenyl-polyethylene glycol, and sirius red were procured from Sigma-Aldrich, Co., USA, whereas thimerosal/merthiolate/sodium ethylmercurithiosalicylate, eosin yellow (Y) stain, and picric acid were procured from Himedia, India. Sildenafil citrate was a gift sample from Watson Pharma. Pvt. Ltd, India. All other chemicals and reagents used were of analytical grade. 

### 2.4. Rho-Kinase 2 (ROCK-II) Inhibition Assay: IC_50_ Determination

*Rho-kinase 2 *(ROCK-II) inhibition assay was performed as per kit protocol and reported earlier [15]. Briefly, 150 nM Y-27632 or 50 *μ*g/mL of herbal extract, 700 nM substrate, and 0.5 ng enzyme were incubated for 10 minutes in a low volume black well plate. The reaction was started by adding 70 *μ*M ATP, and the plate was incubated for 30 min at 37°C. Further, 43.75 nM SA-XL665 and 5 *μ*L of STK Antibody-Cryptate were added and incubated at 25°C for 60 min. HTRF signal was measured by microplate reader, PHERAstar (BMG Labtech, Offenburg, Germany). The enzyme inhibition assay was performed in triplicate, and enzyme inhibition potential of MEBF and Y-27632 was evaluated at different concentrations to determine IC_50_. 

### 2.5. Isolated Rat Corpus Cavernosum Smooth Muscle Relaxation Study

The study was performed as described earlier [15]. Briefly, CCSMs isolated from anesthetized rats were mounted in 4-channel organ bath (Panlab, Spain) containing modified Krebs-Henseleit (K-H) solution maintained at 37 ± 1°C and aerated with carbogen gas (95% O_2_ + 5% CO_2_). CCSMs, connected to a force transducer (Model number TRI201AD; Panlab, Spain) which in turn was attached to a PowerLab/8SP data acquisition system (Chart software, version 7.0; AD Instruments, Australia), were equilibrated for 1 h at 500 mg tension, and K-H solution was replaced every 15 minutes. Relaxant effect of herbal extracts at 0.1, 1, 10, and 100 *μ*g/mL was observed on phenylephrine (3 *μ*M) precontracted CCSMs and was compared with that of sildenafil. Data was acquired by PowerLab/8SP data acquisition system (Chart software, version 7.0; AD Instruments, Australia). 

### 2.6. Sexual Behavior Study

Sexual behavior study was performed as described by Ramachandran et al. [9] with slight modifications. Briefly, sexually active young male rats were divided into 3 groups, each group containing 6 rats, that is, young control (young rats treated with 1% v/v Tween 20), MEBF treated (100 mg/kg of MEBF), and sildenafil treated (5 mg/kg of sildenafil). Similarly, aged male rats were divided into 3 groups, each containing 6 animals, that is, age control (aged rats treated with 1% v/v Tween 20), MEBF treated (aged rats treated with 100 mg/kg of MEBF), and sildenafil treated (aged rats treated with 20 mg/kg of sildenafil). All rats were treated once daily, p.o., for 28 days. Sexual behavior was observed in young as well as aged male rats on 0th, 14th, and 28th days in presence of ovariectomized female rat, brought to estrous phase by administering diethylstilbestrol (1 mg/kg, p.o.) 48 hours prior to and progesterone (5 mg/kg, s.c.) 4 hours before the study. Ovariectomy was performed under anesthesia (ketamine 75 mg/kg body weight and xylazine 10 mg/kg body; i.p.) as suggested by Parhizkar et al. [24]. Sexual behavior study was performed in a wooden cage (45 × 50 × 35 cm) with a glass covering, illuminated with red light after 5 p.m. For the first 5 minutes, male rat was allowed to accustom to the environment, and then a female rat was placed in the cage, and the following sexual behavior parameters were recorded.

 ML (mount latency): time from the introduction of female into the cage of the male up to the first mount.
 IL (intromission latency): time from the introduction of the female up to the first intromission by the male.
 EL (ejaculation latency): time from the first intromission to the ejaculation.
 MF (mount frequency): number of mounts per given period of time.
 IF (intromission frequency): number of intromissions per given period of time.
 PEI (postejaculatory interval): time from end of first ejaculation to start of next intromission.

### 2.7. Sperm Characteristic Study

The effect of vehicle, MEBF, and sildenafil on sperm count, production, and defects was studied as described elsewhere [17–19]. The rats were sacrificed, left caudal epididymis were collected, and contents were transferred to 30 mL of saline solution containing 0.05% triton-X and 0.01% merthiolate (STM solution) in a beaker. After adding 0.5 mL of 1% eosin Y solution, it was mixed well with a glass rod. The volume was made up to 50 mL with distilled water, and solution was again mixed for 3 minutes. Ten *μ*L of this solution was introduced into a clean haemocytometer, and the sperm heads were counted under microscope using 10x objective (Olympus CX41, Olympus, USA) in all the four corner squares. Mean sperm count per epididymis was calculated as mentioned below:

(1) Number  of  sperms  per  caudal  epididymis  =mean  count×50  (total  volume)0.01×0.01  (Volume  of  counting  chamber).

Two hundred sperms were counted in which the live sperms were differentiated by an unstained head and dead sperm as stained head with eosin Y. The live and dead sperm ratio was calculated. 

Defective sperms (bent neck, coiled, tail less, etc.) were also counted, and the results were calculated as given below:

(2) Defective  sperms  percentage  =total  defective  sperms  countednumber  of  sperms  per  caudal  epididymis.

### 2.8. Effect of MEBF on Smooth Muscle and Collagen Level

The effect of vehicle, MEBF, and sildenafil on smooth muscle and collagen: collagen-I and collagen-III was studied using standard methods [20–23]. Penile tissues were excised from anaesthetised rats and fixed in 7% formalin saline for 24 hours followed by washing in distilled water for half an hour. The tissues were then transferred to individual centrifuge tubes containing 70% ethanol in which tissues were stored till processing. The shafts of penes (tissue samples) were dehydrated in graded ethanol for 30 minutes each followed by clearing in chloroform twice for 30 minutes each. The tissues were impregnated in molten paraffin wax bath at 65°C. Tissues were changed thrice (once every 1 hour), and then the tissues were blocked in paraffin. The blocked tissues were allowed to cool down at room temperature. The tissues were then cut into 5 micron thick slices using a microtome (AO Spencer, USA) and placed on glass slides in an oven at 65°C for fixing sections to slide. 

#### 2.8.1. Masson's Trichrome Staining for Smooth Muscle and Collagen

The tissues were hydrated and dipped in coplin staining jar containing Bouin's fluid for 12 hours at room temperature and washed in tap water till sections become white (from yellow) followed by rinsing with distilled water. The tissues were stained in Weigert's iron haematoxylin for 10 minutes (stains nucleus) followed by washing in running tap water and subsequently rinsing it with distilled water. The tissues were then stained in Biebrich scarlet-acid fuchsin staining solution for 5 minutes (stains smooth muscle as red) followed by rinsing it with distilled water. The tissues were treated in phosphomolybdic-tungstic acid solution followed by staining in aniline blue (stains collagen as blue) for 3 minutes. The tissues were then rinsed in distilled water followed by rinsing with dilute acetic acid for 5 minutes. These were further dehydrated in graded alcohol followed by clearing in xylene, and they were then mounted in resinous medium (DPX). The tissues were observed under microscope (Nikon, Japan), and photographs were taken using digital camera (Samsung, Korea).

#### 2.8.2. Picrosirius Red Staining for Collagen-I and -III

Picrosirius red staining for collagen-I and -III of corpus cavernosum was also performed. Briefly, the tissue sections were removed and dewaxed in xylene for 20 minutes and hydrated in graded ethanol and distilled water for 5 minutes each. Nuclei were stained with Weigert's iron haematoxylin for 8 minutes, and then the slides were washed for 10 minutes in running tap water. The sections were stained in picrosirius red (0.1% sirius red in saturated picric acid solution) for one hour followed by washing in two changes of acidified water (5 mL of acetic acid in 1 L distilled water) for 2 minutes each. Water was removed from slides by vigorous shaking, and tissues were dehydrated by 3 changes of 100% ethanol for 10 minutes each. The sections were cleared in xylene and mounted in a resinous medium (DPX). 

The sections were observed under polarized microscope (Nikon, Japan), and photographs were taken using CCD camera (Samsung, Korea).

### 2.9. Phytochemical Profiling

A phytochemical profile of MEBF was also performed to find out the presence of carbohydrates, alkaloids, glycosides, saponins, flavonoids, phenols, and steroids by chemical tests as compiled by Sasidharan et al. and others [25–27]. 

### 2.10. Statistical Analysis

Enzyme inhibition by MEBF was expressed as mean ± standard deviation (SD). Relaxant effects of herbal extract and sildenafil on isolated rat corpus cavernosum and effect of treatment on sexual behavior, sperm characteristic, and penile smooth muscle were expressed as mean ± standard error of mean (SEM). Statistical significance with respect to vehicle was evaluated using one way ANOVA followed by Dunnett's test using SPSS statistics version 17 (SPSS Inc., Chicago, IL, USA). 

## 3. Results 

**Figure 1:**
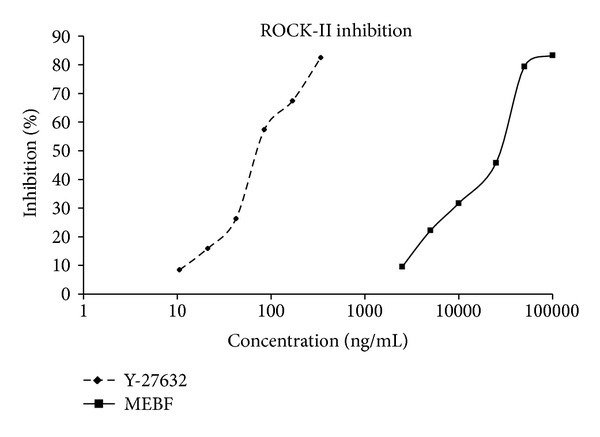
Percent inhibition of ROCK-II by Y-27672 and MEBF at different concentrations. Value is presented as mean ± standard deviation.

The enzyme inhibition assay was standardized using Y-27632 (standard ROCK-II inhibitor), and IC_50_ was found to be 83.74 ± 3.99 ng/mL, while the IC_50_ of MEBF was found to be 20.29 ± 1.83 *μ*g/mL. Percent inhibition of ROCK-II at different concentrations of Y-27632 and MEBF is shown in Figure 1.

**Figure 2:**
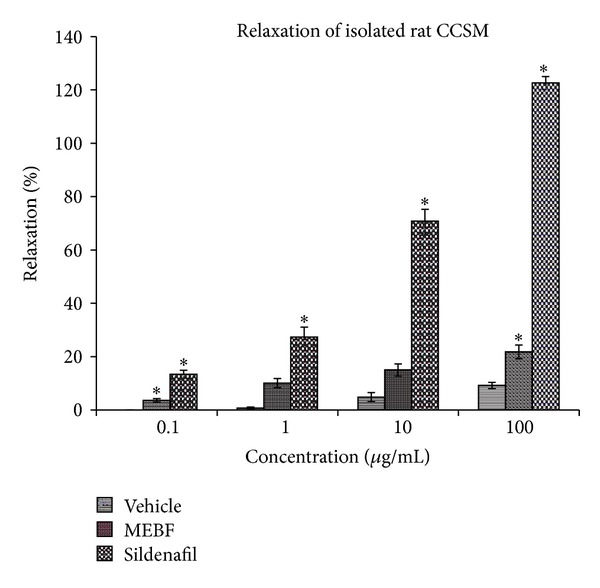
Effect of vehicle, MEBF, and sildenafil on isolated rat corpus cavernosum smooth muscle (CCSM) at different concentrations. *P < 0.05. MEBF/sildenafil versus vehicle. *n* = 6.

The MEBF relaxed CCSM of rat in a dose-dependent manner, with maximum relaxation of 21% being observed at 100 *μ*g/mL. Sildenafil at 100 *μ*g/mL relaxed CCSM beyond 100% (Figure 2). 

We observed that the administration of MEBF increased the sexual behavior of young as well as aged rats but was found to be less potent than sildenafil treatment (Table 1).

The administration of MEBF and sildenafil increased sperm count in young and aged rats, with MEBF being statistically effective in young rats and sildenafil in aged rats. Treatment also increased live sperm percentage and decreased the percentage of defective sperms in both young and aged rats. The effect of MEBF on young rats was statistically significant and is shown in Table 2.

**Figure 3:**
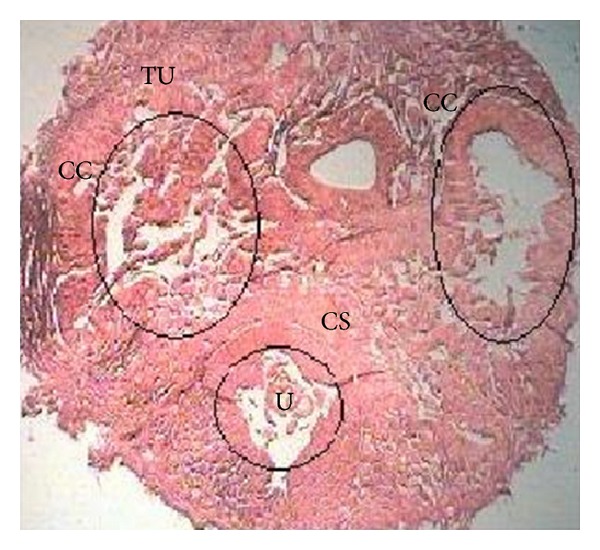
Transverse section (TS) of penile tissue of Wistar rat. TU: tunica albuginea, CC: corpus cavernosum, CS: corpus spongiosum, and U: urethra.

**Figure 4:**
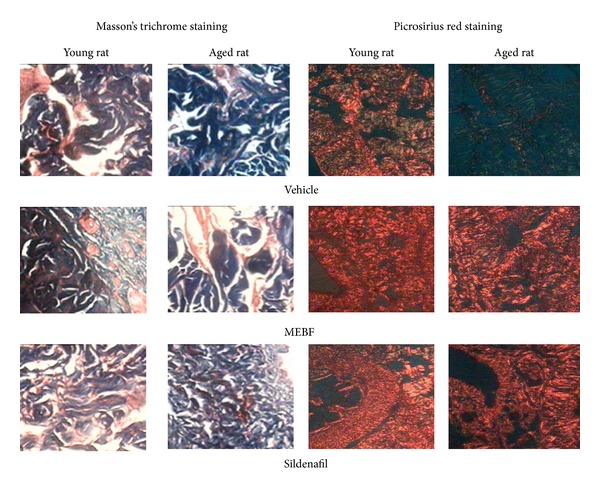
Masson's trichrome staining showing smooth muscle (pink) and collagen (blue) level in CCSM of rat at a magnification of 200x. MEBF treatment increased smooth muscle level in CCSM of young rats, and the effect was better than that of sildenafil. Smooth muscle level decreased in aged rat CCSM, and both MEBF and sildenafil treatment increased smooth muscle level significantly, with the effect of MEBF being more pronounced. Picrosirius red staining showing collagen-III (green) and collagen-I (red orange/yellow) level in CCSM of rat at a magnification of 100x. Collagen-III level was more prominent in CCSM of aged rats in comparison to young rats. Treatment of MEBF and sildenafil decreased collagen-III level.

A transverse section of rat penile tissue exhibiting two corpora cavernosa and one corpus spongiosum covering urethra is shown in Figure 3. In addition, treatment with MEBF and sildenafil increased the smooth muscle content and decreased the level of collagen-I and collagen-III, as evident in Figure 4.

Phytochemical screening of MEBF revealed the presence of carbohydrates, alkaloids, glycosides, saponins, flavonoids, phenols, and steroids which is in agreement with the published literature [26, 27].

## 4. Discussion

The present study was undertaken to identify a possible a mechanism of action for erectogenic and aphrodisiac effects of methanol extract of *Butea frondosa *Koenig ex Roxb. (MEBF) in rats. MEBF not only inhibited ROCK-II, the enzyme implicated in ED, but also relaxed phenylephrine precontracted isolated CCSM of rats significantly. In addition, the extract increased sexual activity/function, improved the quality of sperm production, and increased the level of smooth muscle, while it decreased the collagen level in the rat penile tissue. Most of the changes like decrease in relaxation of CCSM and decline in sexual function are observed in MSD, and reversal of the same may, at least partially, be attributed to the potential of MEBF to inhibit ROCK-II. 

**Figure 5:**
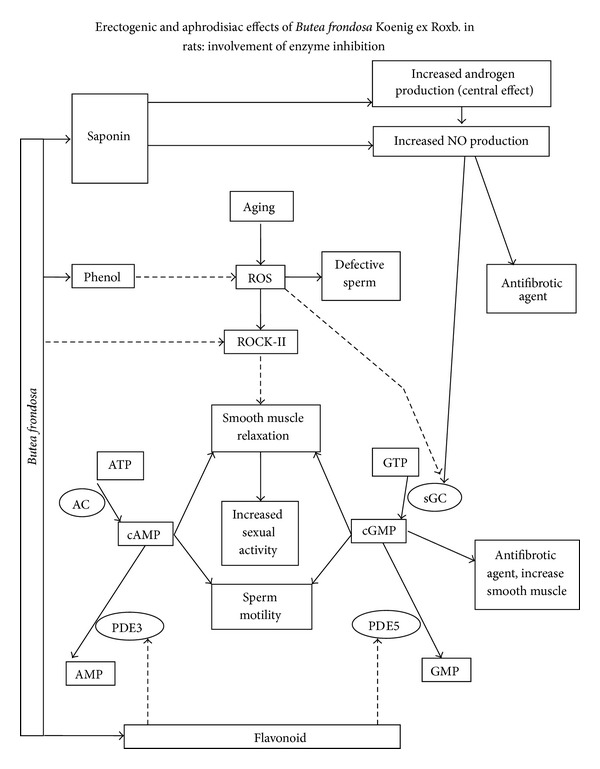
Proposed mechanism of action of MEBF with regard to sexual behavior, sperm characteristics, smooth muscle and collagen level. Solid arrows represent stimulation and dotted arrows represent inhibition.

Penile erection is regulated centrally as well as peripherally. MEBF's property to increase sexual activity/function and relax isolated rat CCSM may be due to a combined effect of both central and peripheral processes. Phytochemical screening of MEBF confirmed the presence of flavonoids [26, 27]. Flavonoid glycosides from methanol extract of *Butea superba*, a plant closely related to *Butea frondosa,* were implicated in inhibition of cAMP specific PDE *in vitro* [28], and aphrodisiac potential of MEBF might be due to PDE inhibition (Figure 5) also. 

Icariin, a flavonoid from *E. brevicornum* Maxim, on administration for four weeks to castrated rats has been reported to increase mRNA and protein expression of neuronal nitric oxide synthase (nNOS) and inducible NOS (iNOS) in CCSM of rats. The treatment also increased intracavernosal pressure (ICP) but did not increase serum testosterone of the castrated rats. Derivatives of icariin were also found to inhibit PDE-5, PDE-6, and cAMP specific PDE [29, 30]. Similarly, protodioscin, a steroidal saponin of *Tribulus terrestris,* was reported to exhibit aphrodisiac property in castrated rats by increasing androgen level [31] which in turn increases nitric oxide (NO) levels in penile tissues by modulating NOS isozymes, that is, endothelial and neuronal NOS [32]. NO relaxes CCSM by increasing cyclic guanosine monophosphate (cGMP) levels, that is, androgen relaxes CCSM through the NO/cGMP pathway. Ginseng saponins have been reported to relax isolated rabbit CCSM by releasing NO from endothelial cells and perivascular nerve [33]. Since saponins were found in MEBF, use of extract might be helpful in the management of ED. 

Oxidative stress is a risk factor for ED, and sildenafil treatment along with antioxidant has been reported to increase erectile function in both rats and humans suffering from ED [34, 35]. Studies have confirmed that methanolic extract of *Butea frondosa* bark possesses 2,2-diphenyl-1-picrylhydrazyl (DPPH), nitric oxide, and superoxide scavenging activity [26]. It has been found that reactive oxygen species (ROSs), responsible for sperm morphological defects [36], activate Rho kinase [37] and reduce the expression of soluble guanyl cyclase/sGC [38], the enzyme that catalyzes conversion of guanosine triphosphate (GTP) to cyclic guanosine monophosphate (cGMP); therefore, the antioxidant potential of MEBF might be useful in the management of ED (Figure 5). 

In young rats, MEBF and sildenafil treatment increased total sperm count as well as live sperm count, while there was a decrease in defective sperm count. However, the statistical significance (*P* < 0.05) was observed only in MEBF treated group. Similarly, in aged rats, MEBF and sildenafil treatment increased total sperm count but it was only significant (*P* < 0.05) for sildenafil treated group. The defects in sperms of aged rats were more in comparison to that of young rats, which could be due to higher oxidative stress related to increase in age. Hence, the effectiveness of MEBF in decreasing defective sperm count may be attributed to antioxidant potential of MEBF. Some studies have suggested that an increase in level of cytosolic cAMP increased sperm motility and sperm viability, whereas an increase in cytosolic cGMP increased sperm motility [39]. In the present study, any observed increase in sperm parameters could also be attributed to cAMP and cGMP specific PDE inhibition. Another hypothesis for increase in sperm count by MEBF and sildenafil treatment is that these pharmacological agents increase blood flow to testes and consequently increase spermatogenesis [40]. 

Sexual function in rats was found to decrease with age. Rats showing normal sexual behavior were selected for the study after initial screening. At 6th month, these rats showed normal sexual activity which decreased slightly at 12th month. At 24th month, 50% (3 out of 6) of the aged rats lost sexual function completely and did not show sexual activity (mounting and intromission) up to 20 minutes after introduction of female rats. This decrease in sexual function could be due to a decrease in smooth muscle level and an increase in collagen level of penile tissue as is evident from histopathological study. Collagen-III level (visible as green mesh under polarized microscope when stained with picrosirius red) also increased in the penile tissue of aged rats and might be responsible for decreased erectile function. In young rats, MEBF treatment increased smooth muscle percentage in rat CCSM. We also observed that the increase in collagen-III level of aged rats decreased after treatment with MEBF and sildenafil. An earlier study reported that chronic treatment with sildenafil, a cGMP specific PDE inhibitor, ameliorated corporal smooth muscle cell loss and fibrosis in 20-month-old rat [20]. In our study, MEBF and sildenafil treatment increased sexual function of all the young rats. Treatment with MEBF and sildenafil also increased sexual function significantly in 50% of the aged rats that had decreased sexual function. The data from 3 aged rats was included for calculation of statistical significance. 

Lastly, antifibrotic properties of MEBF could be due to PDE inhibition and subsequent increase in the level of cGMP, known to have antifibrotic property. In addition, NO has been reported to have antifibrotic property [41], and androgen-induced NO production might be in part responsible for antifibrotic property of MEBF (Figure 5). 

Thus, the findings of the study extended and confirmed the erectogenic potential and aphrodisiac activity of *Butea frondosa* extract. We also propose that the erectogenic and aphrodisiac properties of MEBF could be attributed to its enzyme inhibition potential (ROCK-II and PDE), antioxidant properties, and its potential to increase NO release and androgen levels. Therefore, the extract might be effective in the management of ED, and bioactivity-guided fractionation of the extract in future might lead us to isolate bioactive compounds responsible for the efficacy of the extract. 

## Figures and Tables

**Table 1:** Effect of vehicle, MEBF, and sildenafil on sexual behavior of young and aged rats.

Sexual activities (units)	Young rat (*n* = 6)									Aged rat (*n* = 3)								
	Vehicle			MEBF			Sildenafil			Vehicle			MEBF			Sildenafil		
	0th day	14th day	28th day	0th day	14th day	28th day	0th day	14th day	28th day	0th day	14th day	28th day	0th day	14th day	28th day	0th day	14th day	28th day
ML (seconds)	36.17 ± 1.76	37.17 ± 1.47	37.00 ± 1.03	44.83 ± 4.13	36.83 ± 3.26	27.17 ± 2.74^$^	38.00 ± 2.47	16.83 ± 0.81^#^	11.00 ± 0.61^#^	114.33 ± 7.31	120.00 ± 4.51	122.00 ± 5.03	137.25 ± 6.97	106.75 ± 9.17	81.00 ± 6.18^$^	123.33 ± 8.69	64.00 ± 8.74^$^	37.67 ± 2.33^#^
IL (seconds)	50.33 ± 1.98	48.67 ± 1.75	47.83 ± 1.83	64.00 ± 3.79	50.83 ± 3.18	36.83 ± 2.63^#^	52.83 ± 3.01	21.67 ± 1.04^#^	14.67 ± 0.71^#^	143.33 ± 5.46	148.67 ± 4.26	144.33 ± 7.54	167.00 ± 5.08	131.25 ± 8.50	92.00 ± 6.82^#^	150.33 ± 8.04	76.00 ± 8.33^#^	45.00 ± 4.04^#^
EL (seconds)	327.50 ± 8.71	331.33 ± 7.87	326.17 ± 6.77	305.5 ± 13.89	387.33 ± 19.05*	501.17 ± 25.45^$^	322 ± 6.75	620.5 ± 11.46^#^	899.33 ± 9.26^#^	187.33 ± 8.51	194.67 ± 8.09	190.67 ± 10.33	172.00 ± 9.04	267.50 ± 7.47^#^	355.25 ± 8.80^#^	195.00 ± 9.54	410.67 ± 7.54^#^	383.00 ± 15.13^#^
MF (number)	11.50 ± 0.76	12.00 ± 0.97	12.67 ± 0.97	9.50 ± 0.76	11.83 ± 0.87	14.17 ± 1.11	9.83 ± 0.31	18.50 ± 0.76^#^	23.00 ± 0.82^#^	5.00 ± 0.58	5.00 ± 0.58	5.33 ± 0.33	4.00 ± 0.41	7.00 ± 0.50	10.67 ± 0.76^$^	4.33 ± 0.67	11.67 ± 1.20^#^	15.00 ± 1.00^#^
IF (number)	10.50 ± 0.76	10.17 ± 0.79	10.33 ± 0.95	11.17 ± 0.95	13.17 ± 0.75*	25.00 ± 2.15^#^	11.33 ± 1.25	36.67 ± 0.97^#^	42.83 ± 1.04^#^	4.33 ± 0.88	4.33 ± 0.33	4.00 ± 0.58	3.50 ± 0.65	5.75 ± 0.48	9.75 ± 0.85^#^	3.00 ± 0.58	18.00 ± 0.58^#^	25.00 ± 0.58^#^
PEI (seconds)	230.50 ± 8.97	239.83 ± 8.09	238.67 ± 9.06	275.00 ± 7.14	240.33 ± 9.63	210.67 ± 7.92*	248.83 ± 6.46	210.50 ± 7.48*	172.67 ± 6.51^#^	357.00 ± 11.02	350.33 ± 12.77	354.33 ± 5.84	366.33 ± 17.08	330.75 ± 14.70	300.75 ± 12.47^$^	351.33 ± 10.87	304.33 ± 5.61	267.00 ± 3.46^#^

ML: mount latency, IL: intromission latency, EL: ejaculation latency, MF: mount frequency, IF: intromission frequency, and PEI: postejaculatory interval. *P < 0.05, ^$^ *P* < 0.01, ^#^ *P* ≤ 0.001, methanol extract of *Butea frondosa* (MEBF)/sildenafil versus vehicle (1% Tween 20). *n*: number of animals. All the groups had 6 animals, but 50 percent of aged animals lost sexual function; therefore, those rats were not included for statistical analysis.

**Table 2:** Effect of treatment on sperm characteristics in albino Wistar rats.

Treatment groups	Sperm characteristics		
	Total sperm/g cauda epididymis × 10^7^	Live sperm percent	Defective sperm percent
Young rat			
Vehicle	14.47 ± 1.10	85.17 ± 0.48	9.00 ± 0.44
MEBF	18.62 ± 1.32*	87.33 ± 0.60*	7.02 ± 0.64*
Sildenafil	18.93 ± 0.89	85.50 ± 0.34	7.89 ± 0.30

Aged rat			
Vehicle	12.68 ± 0.71	82.17 ± 1.01	10.25 ± 1.13
MEBF	13.80 ± 0.96	84.50 ± 0.22	8.38 ± 0.59
Sildenafil	16.49 ± 0.60*	84.67 ± 0.71	8.48 ± 0.66

Values are expressed as mean ± SEM; *n* = 6. *P < 0.05, MEBF/sildenafil versus vehicle (1% Tween 20).
